# Draft Genome Sequence of Picalivirus D Recovered from San Francisco Wastewater

**DOI:** 10.1128/genomeA.00654-15

**Published:** 2015-07-02

**Authors:** Alexander L. Greninger, Joseph L. DeRisi

**Affiliations:** a Department of Biochemistry and Biophysics, UCSF, San Francisco, California, USA; b Howard Hughes Medical Institute, UCSF, San Francisco, California, USA

## Abstract

We report here the draft genome sequence of picalivirus D, a member of picalivirus, a picorna-like superfamily, and likely member of the order *Picornavirales*, assembled from metagenomic sequencing of organisms in San Francisco wastewater. This virus likely constitutes a novel genus within the picalivirus family.

## GENOME ANNOUNCEMENT

The picorna-like superfamily is a rapidly expanding taxonomic unit of positive-stranded RNA viruses with conserved RNA-dependent RNA polymerase (RdRp), capsid, and helicase proteins that have a broad host range, including animals, plants, and insects (1–3). Picalivirus is an unassigned member within the picorna-like superfamily that consists of three viral genomes that were originally described in a worldwide survey of sewage enriched for viral particles (4). While originally described as having sequence motif hallmarks of both *Picornavirales* and *Caliciviridae*, picaliviruses increasingly appear to be members of the *Picornavirales* order.

While performing weekly metagenomic sequencing of organisms in San Francisco wastewater, we assembled a 9,570-nucleotide contig, which we are naming picalivirus D. This contig aligned by BLASTx to all three members of picalivirus. Picalivirus D consisted of two open reading frames (ORFs) of 7,023 and 2,238 nucleotides containing the nonstructural and structural proteins, respectively. This genome is 604 nucleotides longer than the complete genome of picalivirus A, which was attained by 5′ and 3′ rapid amplification of cDNA ends (RACE) (4). The RdRp portion of the first polyprotein of picalivirus D demonstrated 31 to 35% amino acid identity to picaliviruses A to C, with 27% amino acid identity to the predicted ABC ATPase/helicase of picalivirus A (the only other picalivirus with a sequence from that region). The capsid polyprotein aligned with 27 to 30% amino acid identity across the entirety of the polyprotein to picaliviruses A to C. The remaining highly ranking BLASTx hits to the picalivirus D genome all derived from the *Picornavirales* order, such as Taura syndrome virus, chicken picornavirus 1, *Aurantiochytrium* single-stranded RNA virus 01, and cricket paralysis virus.

The viral genome was discovered and assembled using PRICE version 1.0, Geneious version 8.0 Assembler, and SURPI version 1.0 from a total of 15,719,690 paired-end 65-bp reads sequenced on an Illumina GAIIx split between these DNAsed and untreated nucleic acid preparations (5, 6). The average coverage of the three contigs using all reads from the sample was 294×.

The original sample was taken from wastewater on 25 January 2010, after a large rainstorm that left >5 inches of rain over the preceding week. Sample processing was performed on 1 liter of wastewater that was concentrated to <5 ml with particles between the sizes of 0.22 μm and 300 kDa using Millipore Pellicon XL 300-kDa filters and 0.22-μm spin columns. The sample was treated with micrococcal nuclease, nucleic acid was extracted using the Zymo viral DNA/RNA kit, and half of the recovered nucleic acid was treated with DNase. Other RNA viruses discovered in this sample include ciliovirus, brinovirus, laverivirus, tombunodavirus, and several other marine RNA viruses and phages (7–9).

### Nucleotide sequence accession number.

The GenBank accession number for picalivirus D is KF478837.
